# Increased Fracture Risk After Bariatric Surgery: a Case-Controlled Study with a Long-Term Follow-Up

**DOI:** 10.1007/s11695-021-05655-9

**Published:** 2021-08-31

**Authors:** Omar Suhail Alsaed, Abdul-Wahab Al-Allaf, Isra Elgenaied, Rawand Abdelnaser Jebril, Sreethish Sasi, Ashraf Omer Ahmed, Rabab Boussarsar, Mohamed Izham Mohamed Ibrahim, Ibrahim Abdulmomen, Wahiba Elhag, Samar A. Al Razaq Alemadi

**Affiliations:** 1 Department of Medicine, Division of Rheumatology, Hamad Medical Corporation, Doha, Qatar; 2 Department of Bariatric and Metabolic Surgery, Hamad Medical Corporation, Doha, Qatar; 3 Pharmacy College of Pharmacy, QU Health Qatar University, Doha, Qatar

**Keywords:** Bariatric surgery, Fracture, Osteoporosis, Obesity, Malabsorption

## Abstract

**Purpose:**

Bariatric surgeries are common procedures due to the high prevalence of obesity. This study aimed to investigate whether bariatric surgery increases fracture risk.

**Material and Methods:**

It was a case-controlled study. Patients who underwent bariatric surgery during 2011 and 2012 were matched for age (± 5 years) and gender to patients on medical weight management during the same period with a ratio of 1:2. The index date was defined as the date of bariatric surgery for both groups. The subject’s electronic medical records were reviewed retrospectively to identify fractures documented by radiology during January 2020.

**Results:**

Randomly selected 403 cases were matched to 806 controls with a median age of 36.0 years (IQR 14.0) and 37.0 years (IQR 14.0), respectively. Seventy per cent of the cohort were females. Eighty per cent received sleeve gastrectomy, and the remaining (17%) underwent gastric bypass. The mean duration of follow-up was 8.6 years. The fracture rate was higher in the surgical group as compared to the controls (9.4% vs 3.5%) with a crude odds ratio of 2.71 (95% CI 1.69–4.36). The median duration for time to fracture was 4.17 years for the surgical group and 6.09 years for controls (p-value = 0.097). The most common site of fractures was feet, followed by hands. Apart from a few wrist fractures, there was no typical osteoporotic sites fracture.

**Conclusion:**

Subjects who underwent bariatric procedures had more non-typical osteoporotic site fractures affecting mainly feet and hands, and fractures tend to occur earlier as compared to controls.

**Graphical abstract:**

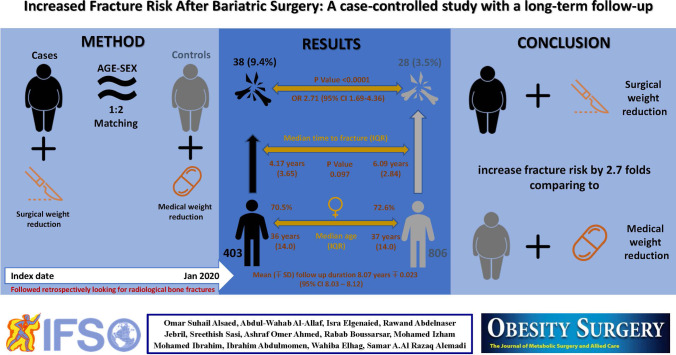

## Introduction

Bariatric surgeries are common procedures with an estimate of > 340,000 operations done in 2011 worldwide [1], and in 2019, around 256,000 bariatric procedures in the USA alone [2]. It is one of the most effective options for achieving significant and durable weight loss. It has short- and long-term complications despite the restricted indications which were updated in 2019 by the American Association of Clinical Endocrinologists, the Obesity Society, and the American Society for Metabolic and Bariatric Surgery (ASMBS) [3].

Bone mineral content loss has been investigated in many studies. A systematic review and meta-analysis revealed that patients who underwent bariatric surgery had significant bone mineral density (BMD) deterioration [4, 5] Most of the bariatric procedures included in these studies were malabsorptive surgeries. BMD loss and fracture post-bariatric procedures have never been estimated in Middle East countries.

The impact of different bariatric procedures on fracture risk was investigated in a few studies; two studies were conducted in the UK population and 4 in North America and Canada. These studies were retrospective, and they differed with the type of bariatric procedures and duration of follow up (Appendix Table 6). The results from these studies were contradictory. The two studies from the UK did not show a statistically significant increment in fracture risk [6, 7]. While in a large, matched cohort study from Canada, fracture risk increased significantly by 1.85-folds in the surgical group [8] and 1.21 (95% CI 1.02–1.43) from a similar study from Taiwan [9]. A meta-analysis of 6 studies published in 2018 showed an increase in fracture risk in all and non-vertebral sites, especially in the upper limbs (RR 1.42, 95% CI 1.08–1.87, and RR 1.68, 95% CI 1.15–2.45). Fracture risk tended to increase in 2 years after surgery but became similar to that of the non-surgical group from years 2 to 5. Notably, only two studies with a mean follow-up of 4.8 years were included in this meta-analysis. The authors of this meta-analysis had suggested having more studies with longer follow-up the duration to investigate the change in fracture risk following bariatric surgery [10]. Another point that urges us to conduct this study results from previous studies was mainly from European and North American populations which cannot be generalized for all populations as it is well known that the mentioned populations have higher fragility fracture rates compared to Asian and African populations [11].

BMD loss and fracture risk post-bariatric procedures have never been estimated in our region or compared with those for the rest of the world. We are expecting that the fracture rate is higher than global figures due to the high prevalence of vitamin D insufficiency in our region, which is 80–90% [12, 13]. However, our study has not designed to answer this question.

The main objective of this study is to investigate whether bariatric surgical intervention increases the risk of fracture. Our study has the advantage of a long follow-up period, and it reflects the situation in the Middle East as most of the previous studies were in the Western.

## Materials and Methods

Subjects who underwent bariatric surgery during 2011 and 2012 were screened for the eligibility criteria until the calculated sample size reached. Patients and controls were selected randomly from this pool during this period. Selected cases were matched with controls for gender and age (± 5 years) in a ratio of 1:2. Controls were selected from the patients’ pool who received non-surgical weight reduction management and followed in the same bariatric clinics. The index date was defined as the date of the bariatric surgery. As our controls do not fitful the criteria to be eligible for surgical intervention, it is difficult to match the two groups with regard to the BMI. Both groups were followed retrospectively from the index date until January 2020 for fracture events as documented by the radiologist.

Data collected from the bariatric and metabolic surgery centre (BMSC) database, which is the principal tertiary centre in Qatar, provides medical and surgical weight management. Bariatric surgical intervention is conducted under ASMBS indications [3]. Cases and controls were randomly selected from those with obesity who underwent medical or surgical weight reduction management during 2011 and 2012 and were included. We took every other patient or control from our electronic lists pool.

Exclusion criteria: patients who are known to have osteoporosis, malignancies, chronic kidney disease, or those who underwent previous gastric banding or gastric balloon, and subjects who did not have clinic visits for more than 2 years from the index date were excluded.

We collected data for all the possible cofounders including exposure to corticosteroid exposure (> 2 prescriptions of systemic corticosteroid after the index date), protein pump inhibitors, and antiepileptics, medical conditions including autoimmune diseases such as systemic lupus erythematosus, rheumatoid arthritis and spondyloarthropathy, primary hyperparathyroidism, inflammatory bowel disease and history of fracture before the index date. Multivariate logistic regression analysis to calculate the adjusted risk ratio was developed.

Our electronic medical records (EMR) are connected to the EMR of the primary healthcare system. Each person in Qatar has a unique healthcare number that carries all medical records throughout his life. For data validation, we did a phone survey for 100 subjects to compare their fracture history with their EMR for fracture events and those were compatible.

### Statistical Analysis

Data from the CERNER® system (patient health record) was input into an Excel sheet. Statistical analyses were done using SPSS 26 for Windows. Data were described using frequency (percentage), mean ± SD, or median (IQR). Kolmogorov–Smirnov test was used to determine the data normality. Chi-square test or Fisher exact test, independent *t* test or Mann Whitney *U*, and one-way analysis of variance (ANOVA) or Kruskal–Wallis test were used for inferential analysis. The risk of fracture was determined using univariate and multivariate logistic regression models. Hosmer and Lemeshow test used to test the overall fit of a model to the observed data. The Cox model for survival data using hazard analysis was applied to assess risk between the interventions. All p values presented were two-tailed and an alpha value of 0.05.

## Results

### Subject Characteristics

Randomly selected 573 potential subjects who received bariatric surgery between 01.01.2011 and 31.12.2012 were screened for the inclusion and exclusion criteria. A total of 170 subjects were excluded as per our exclusion criteria. The remaining 403 cases were matched to 806 controls with a mean (SD) duration of follow-up for both groups was 8.07 $\mp$ 0.023 years (95% CI 8.03–8.12). Both patients and controls were followed for the same period. The subject’s characteristics for both groups are summarized in Table 1. Median age was 36.0 years (IQR 14.0) for surgical group vs 37.0 years (IQR 14.0) for the control group (p = 0.13). No significant differences were found for the gender and age group at > 40 years. As expected the surgical group had a significantly higher baseline median BMI (IQR) 46.93 (7.21) as compared with controls 35.49 (11.9). However, we also found that the surgical group had a higher pre-index date fracture rate (5% vs 1%), proton pump inhibitor uses (41.2% vs 11.9%), primary hyperparathyroidism (0.7% vs 0.0%), and inflammatory bowel disease (0.5% vs 0.0%) as compared with controls, respectively. Sleeve gastrectomy was performed in 83% and gastric bypass in 17%.

**Table 1:** Baseline demographic and clinical data characteristics of both study groups

	Study group		p-value
	Case (n = 403)	Control (n = 806)	
Females (%)	284 (70.5)	586 (72.6)	0.434
**Median (IQR) age, years**	**36.0 (14.0)**	**37.0 (14.0)**	0.013*
Age group > 40, n (%)	126 (31.3)	282 (34.9)	0.202
**Median (IQR) BMI at baseline, Kg/m**^**2**^	**46.93 (7.21)**	**35.49 (11.90)**	< 0.0001*
**Fractures before index date, n (%)**	**20 (5.0)**	**8 (1.0)**	** < 0.0001**
**Systemic corticosteroid, n (%)**	**6 (1.5)**	**37 (4.6)**	**0.006**
**Proton pump inhibitor, n (%)**	**166 (41.2)**	**96 (11.9)**	** < 0.0001**
Antiepileptics, n (%)	7 (1.7)	9 (1.1)	0.372
Spondyloarthropathy, n (%)	1 (0.2)	0 (0)	0.333
**Primary hyperparathyroidism, n (%)**	**3 (0.7)**	**0 (0)**	**0.037**
Rheumatoid arthritis, n (%)	0 (0)	1 (0.1)	0.480
**Inflammatory bowel disease, n (%)**	**2 (0.5)**	**0 (0)**	**0.045**
Systemic lupus erythematosus, n (%)	0 (0)	2 (0.2)	0.317

Chi-square test or Fisher’s exact test was carried out if 25% of the cells have expected count less than 5. *Mann Whitney test

### Fracture Risk Results (Tables 2 and 3)

**Table 2:** Demographic and clinical data characteristics of the fractured patients in both study groups

	**Cases** **(n = 38)**	**Control (n = 28)**	**p value**
Gender n (%)			
Female Male	27 (71.1) 11 (28.9)	23 (82.1) 5 (17.9)	0.299
Median (IQR) age at the time of fracture, years	36.0 (14)	37.0 (14)	0.013
Median (IQR) BMI at baseline, Kg/m^2^	46.9 (7.21)	35.4 (11.11)	< 0.0001
Median (IQR) BMI at time of fracture, Kg/m^2^	36.17 (6.94)	35.98 (11.36)	0.763
Time to the event, median (IQR)	4.17 (3.65)	6.09 (2.84)	0.097

**Table 3:** Anatomical distribution of fracture events for both groups with odd ratio and 95% CI

Item	Case (n = 403)	Control (n = 806)	p value	Odd ratio	Odd ratio 95% CI
**Total fracture events N (%)**	**38 (9.4)**	**28 (3.5)**	** < 0.0001**	**2.71**	**(1.69–4.36)**
Upper limb fractures N (%) Humerus Radio/ulnar Hand	11 (2.7) 1 (0.2) 3 (0.7) 7 (1.7)	13 (1.6) 2 (0.2) 6 (0.7) 5 (0.6)	0.188 0.704 0.623 0.065	1.71 1.00 1.00 2.83	0.76–3.86 0.09–11.07 0.24–4.02 0.89–8.99
Lower limb fractures N (%) Femur Tibia Fibula Foot	25 (6.2) 0 (0) 3 (0.7) 6 (1.5) 16 (4.0)	14 (1.7) 0 (0) 0 (0) 2 (0.2) 12 (1.6)	< 0.001 –- 0.037 0.019 0.011	3.74 –- –- 6.08 2.52	1.92 – 7.28 –- –- 1.22 – 30.27 1.20 – 5.30
Spine fractures N (%)	2 (0.5)	1 (1.0)	0.259	4.02	0.36 – 44.46

Fracture events were significantly higher in the surgical group (38 or 9.4%) as compared with the controls (28 or 3.5%) with an odds ratio of 2.71 (95% CI 1.69–4.36). Numerically, there were more fractures in females (p = 0.299). Fractures occur at a statistically younger age in the surgical group as compared with controls (median IQR age at the time of the fracture was 36.0 $\mp$ 14 years in the surgical group and 37 $\mp$ 14 in controls, p = 0.013). Although the baseline BMI was significantly higher in the surgical group, the BMI at the time of the fractures was not significantly different between the two groups (median IQR BMI at the time of fracture was 36.17 (6.94) years in the surgical vs 35.98 (11.36) years in the controls, p = 0.763). This means that fracture tends to occur earlier in the surgical group than in the controls (median 4.17 years with IQR 3.65 vs 6.09 with IQR 2.84 and p = 0.097). There was no statistically significant difference in the site of the fractures in the two groups.

**Fig. 1:**
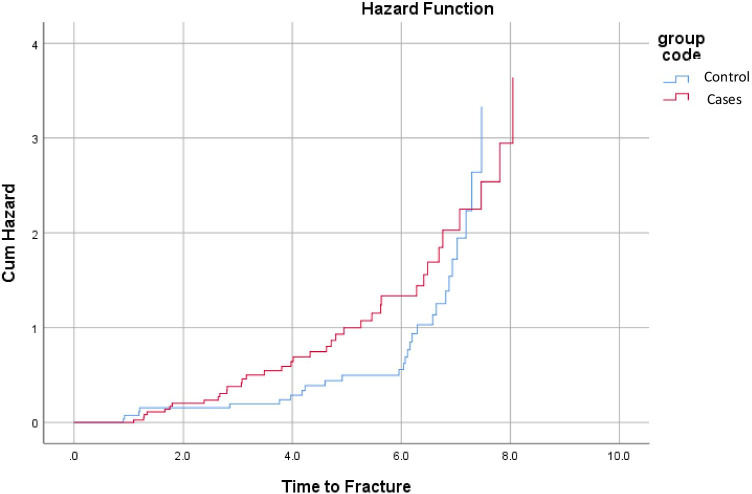
The surgical group (red) developed their fractures much earlier than controls (blue) with the largest gap in cumulative hazard was at 6 years

The time for the highest hazard risk ratio for fracture post-bariatric surgery in comparison with the non-surgical group is around year 6. The cumulative hazard risk between study groups is equal to 7.3 years. Figure 1 illustrates the cumulative hazard risk of fracture between study groups over the study period.

### Adjusted Fracture Risk

In the simple logistic regression (testing for single variable separately), we found that the age group at a cut-off point of 40 years old (p = 0.038), the surgical group (p < 0.0001) if the patient is taking proton pump inhibitor (p = 0.001) and if the patient is taking an antiepileptic medication (p < 0.0001), baseline BMI level ≥ 35 (p = 0.001) and baseline BMI level ≥ 40 (p = 0.001) were found significantly associated with the cases of fracture (Table 4). Patients who were more than 40 years old have a lower odds (OR = 0.592) of association (i.e. 1 patient with the age of more than 40 years old for every 1.7 patients with the age of equal or less than 40 years old). Patients who went through the surgery has almost 3 times more likely to experience a fracture. Among patients who took proton pump inhibitor and antiepileptic medications were around 2 times and 8 times more likely, respectively, to experience a fracture. Further, patients with BMI level of ≥ 35 have a lower odds (OR = 0.274) of association (i.e. 1 patient with the BMI level of ≥ 35 for every approximately 4 patients with the BMI level of less than 35). In addition, patients with a BMI level of ≥ 40 have a lower odds (OR = 0.409) of association (i.e. 1 patient with a BMI level of ≥ 40 for every approximately 2.5 patients with a BMI level of less than 40).

**Table 4:** Association of demographic and clinical data based on fractures incidence in both groups

Confounders	Fracture p-value		OR	95% CI
	Yes (%)			
Female	50 (75.8)	0.473	0.810	0.455–1.443
Male	16 (24.2)	0.473	0.810	0.455–1.443
Age group > 40	30 (45.5)	**0.038**	**0.592**	0.359–0.976
Surgical group	38 (57.6)	** < 0.0001**	**2.896**	1.750–4.793
Systemic corticosteroid	3 (4.5)	0.654	1.314	0.396–4.365
Proton pump inhibitor	237 (20.7)	**0.001**	**2.334**	1.391–3.915
Spondyloarthropathy	1 (0.1)	1.000	0.945	0.933–0.958
Antiepileptic agent	5 (7.6)	** < 0.0001**	**8.443**	2.844–25.062
Systemic lupus erythematosus	2 (0.2)	1.000	0.945	0.933–0.958
Rheumatoid arthritis	1 (0.1)	1.000	0.945	0.933–0.958
Inflammatory bowel disease	2 (0.2)	1.000	0.945	0.933–0.958
Primary hyperparathyroidism	3 (0.3)	1.000	0.945	0.933–0.958
Fractures before surgery	3 (4.5)	0.268	2.131	0.627–7.250
Baseline BMI ≥ 30	63 (100)	0.997	0.000	0.000–na
Baseline BMI ≥ 35	56 (88.9)	0.001	0.274	0.124–0.608
Baseline BMI ≥ 40	42 (66.7)	0.001	0.409	0.239–0.699

The significant variables in the simple logistic regression model are then brought into the multiple regression model, which is the real-life scenario, and confounders were taken into consideration. The results are shown in Table 5.

**Table 5:** Multivariate logistic regression significant predictors and risk factors for fractures

Predictors	B	SE	Wald	df	p-value	Odds ratio	95% CI
Study group	.764	.335	5.192	1	.023	2.147	1.113–4.143
Age above 40	.612	.270	5.140	1	.023	1.844	1.086–3.131
PPI	.426	.293	2.111	1	.146	1.530	0.862–2.717
AEP	1.975	.575	11.796	1	.001	7.206	2.335–22.242
Baseline BMI ≥ 35	− .718	.482	2.222	1	.136	.488	0.190–1.254
Baseline BMI ≥ 40	− .016	.357	.002	1	.965	.984	0.489–1.983

*B* unstandardized regression weight, *SE* standard error, *Wald test* test to determine significant predictor, *df* degree of freedom, *Sig* p value, *Exp(B)* odds ratio

It was indicated that patient’s age above 40, surgical intervention group, and use of antiepileptic are significant predictors and risk factors associated with fractures. Patients who are in the bariatric surgery group have around 2 times more likely to have a fracture if compared to the controls. Patients whose age is above 40 have almost 2 times most likely to have a fracture compared to those below 40 years old. The findings also indicated that patients who are taking antiepileptic agents have around 7 times more likely to have a fracture, but PPI is not a risk factor for fracture.

## Discussion

Firstly, we would like to point to the fact that our study had the highest period for post-bariatric surgical follow up of 8.6 years among the other published studies. In this single-centre case–control study, the risk of fracture in patients with obesity who underwent bariatric surgical intervention, mainly sleeve gastrectomy, was significantly higher at 2.7-folds comparing with age and gender-matched patients with obesity managed with weight reduction therapy. Nevertheless, the vast majority of the fractures were not typical for osteoporotic fragility fracture in both groups. These data pointed to negative sequelae of bariatric procedures on bone health in the long term which needs to be addressed. Post-bariatric surgery data gave no consistent results, but more data is pointing towards increasing the risk of fractures [6–9, 14–16]. These studies differed with the type of bariatric procedures and duration of follow-up (Appendix Table 6 summarized these studies).

As compared to previous studies, our study revealed the highest fracture risk in our surgical group with an OR of 2.71. We think this could be because our surgical intervention group had more history of previous fractures with proton pump and antiepileptics use as compared to controls. However, this could be counteracted by the fact that controls are more likely to be on steroids.

Two UK retrospective studies in 2012 and 2015 did not show a statistically significant increment in fracture risk [6, 7], and we think this could be due to the relatively short follow-up period of 3 years. Lu et al. (2015), from Taiwan, revealed a significantly increased risk of fractures at only 1.2-folds and a slightly longer mean follow-up duration of 4.8 years as compared to the above UK studies. The sub-analysis of this study revealed that the relative risk of fracture is significantly more for the malabsorptive procedure (HR: 1.47, 95% CI 1.01–2.15) and not for the restrictive procedures (HR 1.17, 95% CI 0.97–1.41). However, the study was not powered to show the difference between malabsorptive and restrictive procedures [9].

Rousseau et al. (2016) conducted the largest ever study in this area with 12, 676 patients in the bariatric surgery group and a matched 38, 028 obese patients who undergone a non-surgical intervention and 12, 6760 non-obese patients. Fracture risk increased significantly by 1.8-folds in the surgical group vs 1.13 in the obese non-surgical group in comparison with non-obese subjects [8]. In this study, the mean follow-up duration was only 4.4 years (Appendix Table 6).

We think the longer the duration for the follow-up, the more likely will be the increased risk for the fracture, and this has been demonstrated very well in our study and from the above discussion.

Whether fracture risk differs by the type of bariatric procedure was investigated by Yu et al. (2017), the conclusion was that Roux-en-Y gastric bypass was associated with a 43% (HR1.43, 95% CI 1.13–1.81) increment in risk of non-vertebral fracture compared with adjustable gastric banding [15]. A meta-analysis ran by Zhang et al. (2018) showed a significant increase in non-vertebral fracture risk with RR 1.42. Sub-group analysis showed mixed surgical procedures (mixture of restrictive and malabsorptive procedure) compared with restrictive surgical procedure trended to have a higher fracture risk, but this finding was not statistically significant (RR 1.54, 95% CI 0.96–2.46) [10]. Our strength for the study came from the fact that we have a reasonable number of the patient as calculated by sample size and the long duration of the follow-up (8.6 years). It also came from the Middle East where there is a paucity of data and where the population differs from the Western populations (R1 and R2).

### The Limitations of our Study

The surgical group was not BMI matched to the control group, which is understandable. This is important as it is well known that the higher the BMI, the more the fracture risk [8, 17–19]. In our study, the median BMI at the time of fracture was comparable between the surgical and control groups (36.17 vs 35.98). We also found that our surgical group is more likely to have previous fractures and be on proton pump inhibitors and antiepileptics as compared to controls, and that on contrary, controls are more likely to be on steroids which could affect our results. Smoking, alcohol consumption data, and other comorbidities that can affect bone health were missing in the majority of our patients, which we think could be important cofounders for fracture risk. Sub-clinical vertebral fractures were not included in the outcome, which could underestimate the fracture rate. Most of the fractures in our cohort are not a typical osteoporotic site for fracture; however, these results are pointing to negative sequelae on bone health in general, particularly in the first 4-yearspost-bariatric intervention. This highlights the importance of adherence to post-bariatric procedure recommendations like vitamins, minerals, and proteins supplements and to consider some intervention to counteract such risk. Lastly, we recommend that any future study in this respect should be prospective with consideration of the possible confounders and long enough to answer these important questions.

## Conclusion

Our study was the first study that delineates fracture risk post-bariatric procedure in the Middle Eastern population. The mean duration of follow-up is the longest as compared with previous studies and hence the highest risk for fracture. We also founded the possible confounders, which should be considered in any future study in this field.

Suitable intervention is needed to ameliorate the high risk in the surgical intervention group.

## Appendix

**Table 6:** Summarize studies that investigated fracture risk post-bariatric procedures

Author, year	Methods/matching criteria	Sample size	Procedure	Follow up duration	Fracture risk
Lalmohamed, UK 2012 [7]	Retrospective case-controlled study (age, sex, BMI, practice, year)	Matching ratio 1:6 Cases (2079) Control (10 442)	60% adjustable gastric banding 29% gastric bypass	Mean 2.2	RR: 0.89 95% CI (0.60–1.33)
Nakamura, USA 2014 [14]	Retrospective observational study	258	94% gastric bypass	Mean 8.9	*RR: 2.3 95% CI 1.8–2.8
Lu, Taiwan 2015 [9]	Retrospective case-controlled study (age, sex, Charlson comorbidity index, diabetes, hypertension, hyperlipidemia)	Cases (2064) Control (5027)	86% restrictive procedure 14% malabsorptive procedure	Mean 4.8	RR: 1.21 95% CI 1.02–1.43
Douglas, UK 2015 [6]	Retrospective case-controlled study (age, sex, general practice, and calendar period)	Match ratio: 1:1 Case (3882) Control (3882)	62.9% restrictive procedure 36.6% malabsorptive procedure	Mean 3.4	HR 1.28 95% CI 0.81–2.02
Rousseau, Canada 2016 [8]	Retrospective nested case–control study	Cases (12,676) Obese control (38,028) Non-obese control (126,760)	27.4% Sleeve gastrectomy 41.7% gastric band 9.3% Gastric bypass 21.3% Biliopancreatic diversion	Mean 4.4	RR: 1.85 95% CI 1.68–2.04
Yu, E, USA 2017 [15]	Retrospective case-controlled study (propensity score-matched cohort)	Matching 1:1 Cases: gastric bypass (7516) Control: gastric banding (7516)		Mean 2.3	HR: 1.43 95% CI 1.13–1.81
Fashandi, USA 2018 [16]	Retrospective case-controlled study	Matching 1:1 Cases (1940) Control (1940)	79.4% Gastric bypass 11.2% Gastric banding 7.8% Sleeve gastrectomy	Mean 7.6	p value < 0.000

*Reference was estimated community fracture rate.
